# Drug-Induced Intestinal Angioedema: A Disproportionality Analysis Using the United States Food and Drug Administration Adverse Event Reporting System Database and Literature Review

**DOI:** 10.3390/medsci13040327

**Published:** 2025-12-18

**Authors:** Poovizhi Bharathi Rajaduraivelpandian, Rashmi R. Rao, Ashwin Kamath

**Affiliations:** Department of Pharmacology, Kasturba Medical College Mangalore, Manipal Academy of Higher Education, Manipal, India

**Keywords:** ACE inhibitors, adverse drug reaction, bradykinin, disproportionality analysis, intestinal angioedema

## Abstract

Background: Intestinal angioedema is an important drug-induced adverse effect that is often misdiagnosed due to vague and nonspecific symptoms. This study aimed to identify drugs with potential to cause intestinal angioedema by performing a disproportionality analysis, supplemented with literature review. Methods: Using OpenVigil, we extracted relevant individual case safety reports from the United States Food and Drug Administration Adverse Event Reporting System (FAERS) database. Drugs with signal of disproportionate reporting (SDR) of intestinal angioedema were identified. A literature review was performed using PubMed and Embase databases to identify potential suspect drugs. Results: During 2004–2024, 303 cases of intestinal angioedema were reported to FAERS. Fourteen suspect medications showed SDR; of these, seven drugs were also reported in the literature to have caused intestinal angioedema, including angiotensin-converting enzyme inhibitors, losartan, and acetylsalicyclic acid. A literature search identified 89 relevant articles, providing details of 121 cases. Some drugs linked to intestinal angioedema in the literature did not show SDR. Conclusions: Disproportionality analysis as well as a literature review showed that most patients were middle-aged females on antihypertensive therapy. The results will assist health professionals in determining the temporal association of acute abdomen with the suspected drug, potentially avoiding unnecessary interventions and their attendant complications.

## 1. Introduction

Intestinal angioedema is the oedema occurring within the submucosal space of the bowel that thickens the intestinal wall [1]. Gastrointestinal involvement leads to nonspecific symptoms that consist of nausea, vomiting, and abdominal pain, that can resemble an acute abdominal condition. In rare cases, the massive collection of fluids within the gut lumen, gut wall, and the peritoneum can eventually result in hypovolemic shock [2]. In general, the classification of angioedema is based on its cause, comprising drug-induced; hereditary, with or without a deficiency of the enzyme C1 esterase inhibitor; acquired deficiency of C1 esterase inhibitor; and allergic angioedema [3]. Hereditary angioedema (HAE) affects around one in every 10,000 to 50,000 people, regardless of ethnicity, although a recent Norwegian study suggests that one out of every 100,000 people could be impacted [4]. The epidemiological data of drug-induced intestinal angioedema is undetermined yet. Intestinal angioedema is an exceedingly difficult diagnosis since it is a lesser-known ailment that is not as widespread as angioedema of the tongue, face, genitals, upper airways, or extremities [1]. Reactions to food (that incorporates shellfish, nuts, and some fruits), drugs, bug stings, latex, or other external allergens may culminate in allergic angioedema [3]. HAE attacks can be triggered by physical stress (pressure, mechanical impact), medication (oestrogen contraceptives, angiotensin-converting enzyme inhibitors [ACE-I]), medical interventions, infections, and surgical procedures (like dental surgery), endogenous fluctuations in hormones (menopause, menstruation, pregnancy), or psychological stress [5].

Intestinal angioedema has been reported to be caused by angiotensin-converting enzyme inhibitors (ACE-I) [6]. ACE-I are now the most frequently implicated drugs in the development of iatrogenic intestinal angioedema. ACE-I are frequently prescribed to treat renal and cardiovascular conditions. According to many guidelines, they are one of the first-line medications in treating essential hypertension [7,8]. Plenty of reviews and case reports have established that ACE-Is cause intestinal angioedema [9]. As ACE-Is prevent bradykinin (BK) from being converted to its inactive metabolites, BK builds up and plays a role in the development of intestinal angioedema. Patients merely require supporting care; antihistamines and corticosteroids are not indicated [10,11]. Due to the fact that their nonspecific vague symptoms can mirror an acute abdominal condition and provide diagnostic dilemmas, 57% of patients with ACE-I-induced intestinal angioedema have previously undergone gastrointestinal surgery or biopsies [12]. It would have been curtailed, and symptoms would have improved within 12 to 72 h if ACE-I had been identified as the causal agent and stopped immediately. Therefore, early clinical suspicion of drug-induced intestinal angioedema is crucial for surgeons [6]. A comprehensive analysis of all drugs that have the potential to induce intestinal angioedema is therefore necessary, since many cases may go unreported or undiagnosed, and such rare adverse reactions to drugs are very unlikely to be encountered during clinical trials. There may be other drugs that have the potential to induce intestinal angioedema but have not yet been identified or documented in scientific literature. Updating physicians’ knowledge on drug-induced intestinal angioedema will assist prevent significant morbidity, unnecessary testing, and diagnostic delays [13].

Adverse event databases are useful in identifying drug-adverse event relationships. The United States Food and Drug Administration Adverse Event Reporting System (FAERS) was created to assist the FDA in monitoring the safety of drugs and medicinal products after they have been marketed. FAERS contributes 45% of global adverse event data and serves as one of the biggest pharmacovigilance databases with open public access [14,15]. All drugs with a temporal relation to an adverse drug reaction are reported. It is often difficult to establish the precise causal relationship between a drug and an adverse event, and rescue drugs are often included. Due to this, FAERS is constrained by its very nature, making it impossible to draw definitive conclusions regarding prevalence, incidence, and assessment of causality of adverse drug reactions. Additionally, reporting may be significantly biased depending on regional awareness or national attention [16]. Disproportionality methods are being used to detect signals of disproportionate reporting that may warrant additional clinical exploration to establish the role of the drug in causing an adverse event.

Given that the current literature on drug-induced intestinal angioedema is largely confined to case reports/case series, a comprehensive analysis of spontaneously reported adverse events along with a targeted literature review may reveal the drugs with potential to cause intestinal angioedema. Accordingly, the current study aimed to perform a disproportionality analysis to identify drugs with signals of disproportionate reporting (SDR) of intestinal angioedema. To overcome the inherent limitations of spontaneously reported adverse event data and improve the validity of the findings, the disproportionality analysis has been coupled with literature evidence. This study is aligned to the United Nations Sustainable Development Goal 3.4: “By 2030, reduce by one third premature mortality from non-communicable diseases through prevention and treatment and promote mental health and well-being.”

## 2. Method

### 2.1. Literature Search

We searched the published literature to identify papers describing drug-induced intestinal angioedema. The extracted papers were used to identify the suspect medications, the clinicodemographic characteristics of the patients, outcomes and management, and the proposed mechanism. To identify the relevant literature, we searched the PubMed and Embase databases without any time or language restrictions. All identified case reports were included in the study, irrespective of the quality of the report. The search strategy used was as follows: (“intestinal angioedema” OR “small bowel angioedema” OR “visceral angioedema”). Two authors independently searched for the relevant studies, and the results were deduplicated.

### 2.2. ADR Database Analysis

Since not all cases of potentially drug-induced intestinal angioedema may be reported in the literature, we supplemented the literature search by identifying relevant adverse event reports from the United States Food and Drug Administration Adverse Event Reporting System (FAERS) database, a database of spontaneously reported adverse events by consumers, doctors, other healthcare professionals, and lawyers. All individual case safety reports (ICSRs) reported to the FAERS from the first quarter of 2004 to third quarter of 2024 were included. The FAERS data was accessed using the OpenVigil (version 2.1) software application [17]. OpenVigil provides an intuitive custom user interface for drug/ADR search and for conducting disproportionality analysis. Cases of potentially drug-induced intestinal angioedema were identified by searching for the following Medical Dictionary of Regulatory Activities (MedDRA) low level terms (LTs): intestinal angioedema and small bowel angioedema. ICSRs reported from United States involving all ages, genders, indications, outcomes, and reporter types were included for the analysis. The drugs listed as the suspect medication (primary or secondary) in one or more reports were considered to have caused the adverse event. To determine whether the observed reporting rate for the event is higher than expected for a particular drug, disproportionality analysis was performed using reporting odds ratio (ROR) [18]. A ROR >2 with the lower end of the 95% confidence interval >1 and a minimum of 3 reported cases was considered as SDR.

Among the drugs with SDR, those that have not yet been described in the literature as having caused intestinal angioedema were identified. A literature search was performed again using the PubMed and Embase databases by prefixing the drug name to the search string (“drugname” AND (“intestinal angioedema” OR “small bowel angioedema” OR “visceral angioedema”)). If no relevant literature was obtained on searching these databases, we searched the first three pages of search results in Google Scholar.

The study protocol was approved by the Institutional Ethics Committee. The study adheres to the READUS-PV (Reporting of a disproportionality analysis for drug safety signal detection using ICSRs in pharmacovigilance) guideline.

## 3. Results

### 3.1. Clinical Presentation of Patients with Intestinal Angioedema Based on Literature

A literature search yielded 250 articles, 94 in PubMed and 156 in Embase. After removing duplicates, 177 articles were available. Screening of the title and abstract resulted in 89 relevant articles, which provided details of 121 cases. Of these, age was reported for 117 patients (Supplementary Table S1). The median age of the patients was 48 years (interquartile range, 42–58 years; range, 19–92 years); 70.09% (82/117) were females. The most common indication for drug use was hypertension (72.65% [85/117]) with or without other comorbidities. Regarding time to onset after initiation of treatment with the suspect drug, 46.84% (37/79) cases had onset in less than a month, 20.25% (16/79) cases had onset between 1 month to 1 year, and in 32.91% (26/79) cases, onset was after 1 year or more. In 5 cases, the time to onset was unclear, and no data was available in 37 cases. Regarding time to resolution, it was within a day in 60.61% (40/66) cases, and between one day and one week in 39.39% (26/66) cases. No data was available in 36 cases.

The details of the cases with suspected ACE-I-induced angioedema are presented in Table 1 and Supplementary Table S1. Of the 86 ACE inhibitor-related case reports, lisinopril was the most commonly implicated ACE-I (65.12%, 56/86). The discontinuation of the suspect medication was the only measure taken in 29 cases, without any additional treatment or management. ADR was frequently managed by administration of intravenous fluids (19 cases) and supportive care (14 cases). In four case reports, patients underwent unnecessary surgical interventions, such as exploratory laparotomy (suspected intestinal ischemia), cholecystectomy, and unspecified surgical interventions, as diagnosis was delayed without getting any relief from symptoms. The concomitant medications reported were diuretics (17 cases), statins (11 cases), acetylsalicylic acid (9 cases), amlodipine (6 cases), and metformin (6 cases). No similar past history and multiple past similar history was noted in 67 (83%) and 20 (24%) case reports, respectively. Past failed exploratory laparotomy and cholecystectomy were reported in two case reports. Positive rechallenge was noted in 5 out of 82 case reports (6%).

Table 2 and Supplementary Table S2 present the details of cases of intestinal angioedema suspected to be induced by drugs other than ACE-Is. Among the 23 case reports, nonionic contrast medium was the most implicated drug. The discontinuation of the suspect medication was the only measure taken in 13 cases, without any additional treatment or management. ADR was frequently managed by hydration (5 cases) and bowel rest (4 cases). In one report of a patient who underwent injections of hyaluronic acid–based dermal fillers in the face and developed intestinal angioedema, the episode was successfully treated with injection hyaluronidase. No similar past history and multiple past similar history was noted in 17 (81%) and 6 case reports (19%), respectively. Positive rechallenge was noted in 3 out of 23 case reports (13%).

### 3.2. Disproportionality Analysis

**Figure 1:**
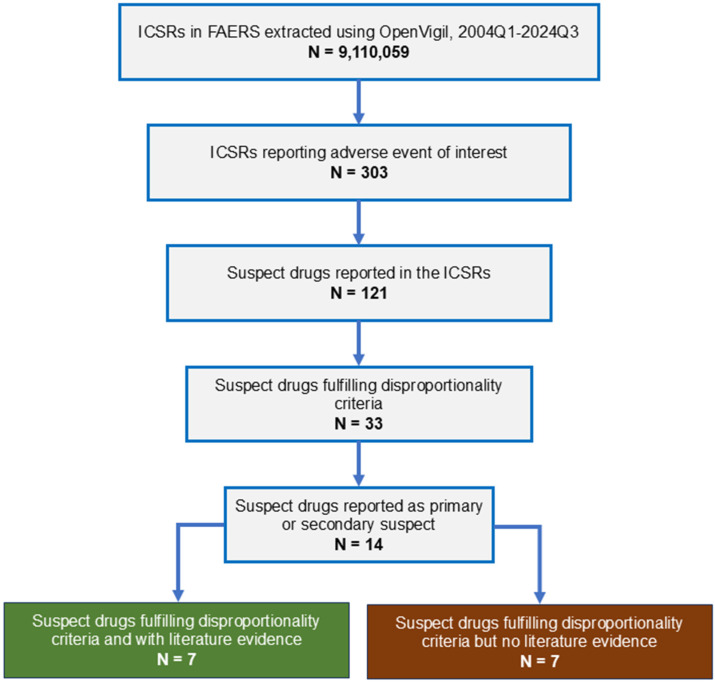
Study flow diagram. ICSR, individual case safety report; FAERS, Food and Drug Administration Adverse Event Reporting System.

From 2004 to 2024, 9,110,059 adverse events were reported to FAERS (Figure 1). Three hundred and three cases of intestinal angioedema were reported. Age was reported in 277 ICSRs; the median age of the patients was 49 years (interquartile range, 41–61 years; range, 23–87 years); gender was reported in 282 ICSRs; 75.18% (212/282) were females. The most common indication for drug use was hypertension (72.69% [189/260]). None of the cases resulted in death.

Thirty-three medications showed SDR for intestinal angioedema. Of these, 14 drugs were reported as primary or secondary suspect medications in one or more ICSRs. The disproportionality results for these medications are shown in Table 3. As seen, the maximum number of ICSRs were reported with lisinopril (254) followed by losartan and hydrochlorothiazide (38 each), pantoprazole (21), amlodipine (17), and nifedipine (15).

Of the 14 suspect medications, case reports were available for 7 drugs, implicating them in the causation of intestinal angioedema (Table 4). Four of these were ACE-Is, and one was an angiotensin receptor blocker. The drugs for which supporting literature was not available were amlodipine, atorvastatin, clonidine, dicyclomine, metformin, nifedipine, and nisoldipine. However, some of these, such as amlodipine, atorvastatin, and metformin, were commonly reported as concomitant medications in the case reports.

## 4. Discussion

Our study described the clinicodemographic profile of patients with suspected drug-induced intestinal angioedema and identified the potential causative drugs. The age and gender presentation as determined by the literature review and FAERS database analysis closely match each other, with most patients being middle-aged females with hypertension. Besides ACE-Is, particularly lisinopril, losartan, and acetylsalicylic acid are the other drugs with evidence both from disproportionality analysis and literature review. Of note, some drugs implicated in causing intestinal angioedema based on case reports did not show SDR; similarly, many drugs with SDR did not have supporting literature evidence. A number of the later drugs, although mentioned as suspect drugs in one or more ICSRs, were more often reported as concomitant medications, and therefore are likely to be false positives. Excluding the names of the 4 suspect drug groups (ACE-I, calcium channel blockers, hormone replacement therapy, nonionic contrast medium), literature review identified 19 unique drugs (Table 4) which potentially induced intestinal angioedema. Of these, hydrochlorothiazide and indapamide were reported in combination with lisinopril and perindopril, respectively. Of these, 7 drugs showed SDR on disproportionality analysis. However, 13 drugs, including hormone replacement therapy, did not appear in the disproportionality analysis results. Barring sirolimus, tacrolimus, and hyaluronic acid, the rest of the drugs were ACE-Is or angiotensin receptor blockers, or nonionic radiocontrast media. It is to be noted that ACE-Is and estrogen-containing medications, which have been reported to cause intestinal angioedema, are also known triggers for HAE attacks in susceptible patients [5].

The mechanisms leading to the development of angioedema, in general, include excess production or inability to degrade the vasoactive compounds like histamine, BK, and leukotrienes, resulting in increased vascular permeability [11]. Drug-induced angioedema can be of two types, allergic and nonallergic. Histamine, which is involved in type 1 IgE hypersensitivity reaction, is the initiating factor in allergic angioedema [114]. The most commonly implicated drugs are beta-lactam antibiotics, quinolones, and the iodinated contrast media [115]. NSAIDs and ACEIs are the commonly encountered drugs implicated in nonallergic angioedema [116]. The primary underlying mechanism with these drugs is interference with arachidonic acid metabolism by blocking cyclooxygenase (COX) enzyme, increased production, and decreased metabolism of BK and C1 esterase inhibitor deficiency [115].

Histamine-mediated angioedema is a type 1 hypersensitivity reaction. Certain iodine and gadolinium-based contrast media can cause histamine-mediated angioedema by direct degranulation of mast cells and basophils [117,118]. It is also proposed that inhibition of COX enzyme by NSAIDs in arachidonic acid pathway leads to increased synthesis of leukotrienes, which increase vascular permeability and induce hyperresponsiveness to histamine [119,120].

**Figure 2:**
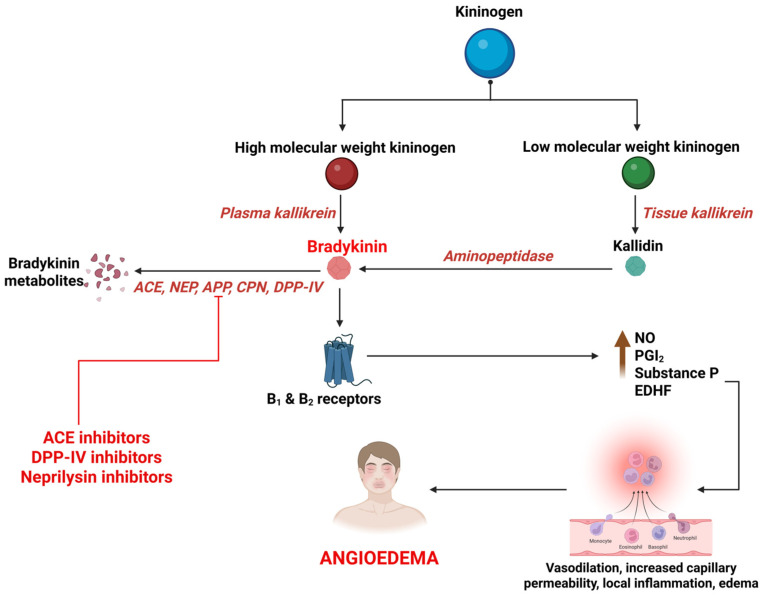
Synthesis and breakdown of bradykinin and its association with drug-induced angioedema. B_1_, bradykinin receptor type 1; B_2_, bradykinin receptor type 2; NO, nitric oxide; PGI_2_, prostacyclin; EDHF, endothelium-derived hyperpolarizing factor; ACE, angiotensin converting enzyme; NEP, neutral endopeptidase; APP, aminopeptidase P; CPN, carboxypeptidase N; DPP-IV, dipeptidyl peptidase IV.

BK is a potent, short-acting vasoactive peptide that mediates inflammation and vasodilation in multiple signaling cascades. It is synthesized primarily through the activation of the kallikrein–kininogen system (KKS), by a protease called kallikrein [121,122]. BK then acts on BK receptor type 1 and 2 to mediate various actions that constitute the regulation of vascular tone, maintenance of kidney function, and defense against ischemic reperfusion damage. It also plays an important role in inflammation by releasing nitric oxide and prostaglandins, causing angioedema by increase in blood vessel permeability and diffusion of plasma into submucosal tissues [123,124,125,126] (Figure 2). BK is primarily degraded by Angiotensin Converting Enzyme (ACE), neutral endopeptidase (NEP), aminopeptidase P (APP), carboxypeptidase N (CPN), dipeptidyl peptidase IV (DPP-IV), and Kininase I. The primary enzyme, ACE (Kininase II), degrades BK to inactive metabolites. The ACEIs interfere with this process, leading to accumulation of BK. The metabolism of BK is taken over by secondary enzymes; deficiencies of these further interfere with the process of BK degradation, extending its action and culminating in angioedema [13,127] (Figure 2).

The exact mechanism for ARB-induced angioedema is not clearly understood. There are two postulated mechanisms in the literature. First, the ARBs exert a feedback rise in plasma levels of angiotensin II by suppressing the Angiotensin II type 1 (AT 1) receptor, and this causes self-inhibition of the ACE, causing BK accumulation and angioedema [10]. Second, ARBs block the AT 1 receptor and increase the angiotensin II levels, which then activates the AT 2 receptor and causes release of BK, resulting in angioedema [128].

The drug-induced angioedema linked to prostaglandin and leukotrienes occurs with NSAIDs. It’s a non-immunological reaction that involves the cyclooxygenase (COX) pathway. The NSAIDs interfere with the metabolism of arachidonic acid through the inhibition of COX 1 and 2 pathways, which leads to a decrease in prostaglandin synthesis and loss of its beneficial effects and redirects the arachidonic acid towards the 5-lipoxygenase pathway. This leads to the release of cysteinyl leukotrienes, which is a vasoactive substance, causing an increase in vascular permeability and edema, resulting in the development of angioedema. Though the primary mechanism for NSAID-induced angioedema is COX inhibition, mast cell and basophil degranulation that release chemical mediators like histamine can contribute to the above mechanism [115,129].

The importance of this study lies in the fact that many cases of drug-induced intestinal angioedema experienced significant delays in diagnosis and underwent unnecessary diagnostic or therapeutic interventions, including surgeries. The presence of cases of multiple episodes of positive rechallenge indicates the lack of adequate awareness among the treating physicians. Since there is evidence that most of the patients experienced complete resolution following discontinuation of the suspect drug with or without supportive measures, having a list of potential suspect medication will aid in proper management of the condition.

The strength of our study is that we systematically combined the disproportionality analysis approach with literature review to overcome the limitations associated with FAERS data, in particular the presence of false positives. The similarity in the clinicodemographic profiles of the patients from FAERS and the published literature strengthens the observation that drug-induced intestinal angioedema predominantly affects middle-aged females. However, the study also has limitations. While we supplemented adverse event database analysis with literature review, given that intestinal angioedema is not very common and difficult to diagnose without an index of suspicion, significant underreporting of cases cannot be ruled out. This is more likely to impact drugs other than ACE-Is, which are not known to cause the event. Another important limitation is the potential for reporting bias, where medications may be incorrectly classified as suspect or concomitant. This can lead to false associations or missed signals. For instance, commonly co-prescribed drugs such as amlodipine and metformin were frequently listed as concomitant medications in case reports, yet showed signals of disproportionate reporting, raising the possibility of confounding. These medications are commonly prescribed alongside antihypertensives, particularly ACE inhibitors, which are known to cause intestinal angioedema. Their frequent co-prescription likely contributes to their appearance in FAERS reports. Importantly, no case reports were found implicating these drugs as causative agents, and they were often listed as concomitant medications. This highlights the need for cautious interpretation of SDRs and underscores the importance of integrating literature evidence to validate pharmacovigilance signals. We studied a single adverse event database. Inclusion of other international databases, such as EudraVigilance or VigiBase, could provide a broader perspective and potentially identify additional signals. However, differences in coding practices, drug availability, and reporting behaviors across regions may introduce heterogeneity and bias, complicating direct comparisons. Future studies incorporating multiple pharmacovigilance databases could help validate the findings and improve generalizability.

## 5. Conclusions

The available evidence suggests that in the vast majority of cases of drug-induced intestinal angioedema, the diagnosis was often delayed until after several similar episodes. Antibiotics and emergency surgery have been attempted in multiple instances, with unsuccessful outcomes. Symptoms appear immediately after administration of contrast medium or within a month to a year after starting other drugs. Discontinuation of the suspect drug enabled the complete recovery of all patients within hours to a few days. Literature review and adverse event database evidence reveal that the causative drugs, besides the commonly reported ACE-Is, particularly lisinopril, are angiotensin receptor blockers and acetylsalicylic acid. Some drugs, such as oestrogen, radiocontrast media, and immunosuppressants, are potential suspect medications despite the absence of evidence from disproportionality analysis.

The results of this study will assist health professionals in determining the temporal association of acute abdomen with the suspected drug, thereby potentially avoiding unnecessary interventions and their attendant complications. Given the diagnostic challenges and frequent misattribution of symptoms to surgical or infectious causes, clinicians should maintain a high index of suspicion for drug-induced intestinal angioedema, especially in patients presenting with recurrent abdominal pain and a history of ACE inhibitor or ARB use. Early recognition can prevent unnecessary imaging, antibiotic therapy, and surgical procedures such as exploratory laparotomy. Prescribers should consider discontinuation of the suspect drug as a first-line response when intestinal angioedema is suspected, and monitor for rapid symptom resolution. Incorporating awareness of this adverse event into routine clinical assessment may improve diagnostic accuracy and reduce patient morbidity.

## Figures and Tables

**Table 1:** Summary of case reports of ACE-I induced intestinal angioedema.

Suspect Drug	Age (in Years), Gender (M:F)	Indication (Number of Reports)	Symptoms	Past History of Angioedema	Outcome	Reference
Captopril	56, 2:0	Hypertension (2) Coronary artery disease (1) Congestive heart failure (1)	Sudden swelling of lips, face, and tongue Nausea and emesis Abdominal pain (generalised) Diarrhoea	Present: 0 Absent: 2	All recovered	[19,20,21]
Enalapril	51, 1:10	Hypertension (11) Coronary artery disease (1) Congestive heart failure (1) Idiopathic Collapsing Glomerulopathy (1) Hypertension (Related to Calcineurin Inhibitors) (1)	Abdominal pain (intermittent, severe, recurrent, acute, diffuse, generalized) Vomiting (including bilious vomitus) Diarrhoea Nausea Bloating Heartburn Dizziness General malaise	Present: 5 Absent: 6	All recovered	[22,23,24,25,26,27,28,29,30,31,32]
Fosinopril	67, 0:1	Hypertension (1)	Severe mid-epigastric pain associated with nausea and vomiting	Present: 1 Absent: 0	Recovered	[33]
Lisinopril	49, 11:42	Hypertension (48) Dilated Cardiomyopathy (1) Chronic kidney disease, Hypertension (1) Proteinuria (1) Following cerebral infarction (1)	Nausea Vomiting (including emesis, bilious, non-bloody, non-bilious, intractable, Coffee-colored) Diarrhoea (watery, chronic, non-bloody) Bloating and abdominal distension Hypotension and tachycardia Lightheadedness and dizziness Dyspnoea Heartburn Oropharyngeal edema Small bowel obstruction Subjective fevers and chills	Present: 12 Absent: 41	Recovered: 54 Not mentioned: 2	[9,12,33,34,35,36,37,38,39,40,41,42,43,44,45,46,47,48,49,50,51,52,53,54,55,56,57,58,59,60,61,62,63,64,65,66,67,68,69,70,71,72,73,74,75,76,77,78,79,80,81,82,83]
Lisinopril + Valsartan	44, 0:1	Hypertension (1)	Acute onset of abdominal pain	Present: 0 Absent: 1	Recovered	[82]
Perindopril	43, 3:5	Hypertension (8)	Abdominal pain (sudden onset, severe, diffuse, periumbilical, lower abdominal, intermittent cramps) Vomiting (including bilious) Nausea Diarrhoea (including non-bloody) Abdominal distension and bloating Urge to have bowel movement and difficulty passing stool	Present: 0 Absent: 8	All recovered	[6,84,85,86,87,88,89,90]
Ramipril	68, 0:3	Concurrent Coronary and hypertensive left ventricular dysfunction (1) Hypertension (1) Hypertensive cardiomyopathy (1)	Abdominal pain (intractable, diffuse, crampy, progressive) Nausea and vomiting Hoarse, raspy voice Difficulty breathing	Present: 0 Absent: 3	All recovered	[91,92,93]
Benazepril	55, 2:2	Hypertension (4)	Oropharyngeal edema and gargled speech Colicky abdominal pain Abdominal pain (intermittent, dull ache) Nausea and vomiting (including bilious, non-projectile) Bloating	Present: 0 Absent: 4	All recovered	[94,95,96,97]
Angiotensin-converting enzyme inhibitor	34, 0:1	Background of renal transplant for medullary cystic kidney disease (1)	Stereotyped episodes of abdominal pain with loose stools	Present: 1 Absent: 0	Recovered	[98]

**Table 2:** Summary of case reports of intestinal angioedema induced by drugs other than angiotensin-converting enzyme inhibitors.

Suspect Drug	Age (in Years), Gender (M:F)	Indication (Number of Reports)	Symptoms	Past History of Angioedema	Outcome	References
Intravenous nonionic iodinated contrast media (Iopamidol, Iohexol)	51, 7:1	Abdominal CT (4) Abdominopelvic CT (4)	GI symptoms, mild abdominal discomfort, vomiting	Absent	All recovered	[99,100,101]
Gadobenate dimeglumine	34, 1:0	Contrast agents for imaging	Abdominal cramps	Absent	Recovered	[102]
Diatrizoate meglumine and diatrizoate sodium solution intravenous, iopromide	46, 3:0	Abdominal CT	Mild abdominal discomfort	Absent	All recovered	[103]
Angiotensin receptor blockers (Losartan, Irbesartan)	58, 2:4	Hypertension (4) Hypertension with renal disease (1), Not specified (1)	Abdominal pain, diarrhea	Present (2)	All recovered.	[13,22,104,105,106,107]
Hormone replacement therapy	58, 0:1	Hysterectomy	Abdominal pain, nausea, vomiting	Present	Recovered	[108]
Acetylsalicylic acid	80, 0:1	Lumbago	Acute epigastric pain, nausea, vomiting	Present	Recovered	[109]
Calcium channel blocker	56, 0:1	Not specified	Abdominal pain, nausea, vomiting, diarrhea	Present	Recovered	[110]
Hyaluronic acid–based dermal fillers	47, 0:1	Not specified	Abdominal pain	Present	Recovered	[111]
Sirolimus	38, 0:1	Renal transplantation	Abdominal pain, vomiting, diarrhea	Absent	Recovered	[112]
Tacrolimus	19, 0:1	Orthotopic kidney transplantation	Abdominal pain, diarrhea	Absent	Recovered	[113]

**Table 3:** Suspect drugs showing signals of disproportionate reporting for intestinal angioedema with or without evidence from literature review.

Suspect Drug	Reporting Odds Ratio (ROR)	ROR CI Lower Bound	ROR CI Upper Bound	Drug of Interest + AE of Interest	Other Drugs + AE of Interest	Drug of Interest + Other AEs	Other Drugs + Other AEs
**With literature evidence**							
Acetylsalicylic acid	3.19	1.87	5.45	14	289	136,271	8,973,485
Benazepril	11.66	4.82	28.22	5	298	13,090	9,096,666
Captopril	59.96	19.21	187.18	3	300	1519	9,108,237
Enalapril	16.64	7.41	37.35	6	297	11,046	9,098,710
Hydrochlorothiazide	14.39	10.24	20.22	38	265	89,884	9,019,872
Lisinopril	350.55	258.17	475.97	254	49	132,746	8,977,010
Losartan	19.49	13.87	27.38	38	265	66,537	9,043,219
**Without literature evidence**							
Amlodipine	4.56	2.79	7.43	17	286	117,344	8,992,412
Atorvastatin	3.25	1.83	5.79	12	291	114,112	8,995,644
Clonidine	16.28	9.14	29.00	12	291	23,022	9,086,734
Dicyclomine	12.83	4.12	40.03	3	300	7093	9,102,663
Metformin	2.15	1.18	3.93	11	292	156,880	8,952,876
Nifedipine	34.38	20.45	57.79	15	288	13,781	9,095,975
Nisoldipine	183.29	58.58	573.46	3	300	497	9,109,259

AE, adverse event; CI, confidence interval.

**Table 4:** Drugs with potential to cause intestinal angioedema as identified by literature search with or without signal of disproportionate reporting (SDR).

Suspect Drugs with SDR on Disproportionality Analysis *	Suspect Drugs Without SDR on Disproportionality Analysis *
Acetylsalicylic acid	Diatrizoate/Iopromide
Benazepril	Fosinopril
Captopril	Gadobenate Dimeglumine
Enalapril	Hyaluronic Acid
Lisinopril	Iohexol
Lisinopril/Hydrochlorothiazide	Iopamidol
Losartan	Irbesartan
	Perindopril
	Perindopril/Indapamide
	Ramipril
	Sirolimus
	Tacrolimus

* Excludes drug groups—angiotensin-converting enzyme inhibitors, calcium channel blockers, hormone replacement therapy, nonionic contrast medium.

## Data Availability

The original contributions presented in this study are included in the article/supplementary material. Further inquiries can be directed to the corresponding author. These data were derived from the following resource available in the public domain: United States Food and Drug Administration Adverse Event Reporting System (FAERS) (https://www.fda.gov/drugs/surveillance/fdas-adverse-event-reporting-system-faers (accessed on 5 September 2025)).

## Supplementary Materials

The following supporting information can be downloaded at: https://www.mdpi.com/article/10.3390/medsci13040327/s1.
