# Supplementary material for: Drug-Induced Intestinal Angioedema: A Disproportionality Analysis Using the United States Food and Drug Administration Adverse Event Reporting System Database and Literature Review
Source: Med Sci (Basel). 2025 Dec 18;13(4):327. doi: 10.3390/medsci13040327 (PMC12735144; doi:10.3390/medsci13040327)
Supplement: Supplementary file 1 [file medsci-13-00327-s001.zip › Supplementary Table S1.pdf]

Supplementary Table S1. Case reports of ACE-I induced intestinal angioedema.

Study	Age (in Years) and Gender	Suspect Drug and Regimen	Indication(s)	Concomitant Medication (s)	Concomitant Medication Indication(s)	Symptoms	Past History of Angioedema	ADR Management	Outcome
Myslinski et al. Case 1[1]	62, M	Lisinopril	Hypertension	Amlodipine, chlorthalidone, atorvastatin, allopurinol, acetylsalicylic acid, and omeprazole	Obesity, cardiac arrest due to presumed ventricular tachycardia, hypertension, gout, and sleep apnoea	Abdominal pain, vomiting, diarrhoea, lightheaded, dizzy, hypotension	Similar seven episodes in the past 3 years responded to intravenous fluids and recovered within 72 hours	Lisinopril was discontinued, 2000 cc of normal saline, 2 L/min oxygen, ondansetron	Recovered
Myslinski et al. Case 2[1]	33, F	Lisinopril	Hypertension	Amlodipine, furosemide, carvedilol, triamterene/hydrochlorothiazide, pravastatin, and metformin.	Hypertension, non-insulin-dependent diabetes, and nonischaemic cardiomyopathy	Abdominal pain, vomiting, tachycardia	Nil	Lisinopril was discontinued, empiric antibiotics for 24 hours, intravenous fluids.	Recovered
Orr et al.[2]	72, F	Enalapril 20 mg/day	Hypertension	Oestrogen replacement therapy, lovastatin	Not mentioned	Intermittent abdominal pain, vomiting	Facial swelling recovered in a week after discontinuing enalapril.	Enalapril was discontinued	Recovered
Salloum et al.[3]	55, M	Perindopril 4 mg/day	Hypertension	Indapamide	Nil	Sudden onset periumbilical and left iliac fossa pain with vomiting	No	Perindopril was discontinued, symptomatic treatment	Recovered
Gillion et al.[4]	41, F	Enalapril 20 mg/day	Idiopathic collapsing glomerulopathy	Tacrolimus, furosemide, spironolactone, and lercanidipine	Idiopathic collapsing glomerulopathy	Severe abdominal pain	Five similar episodes in the past 3 years	Enalapril was discontinued	Recovered
Dietler et al.[5]	92, F	Ramipril	Concurrent coronary and hyperten	Acetylsalicylic acidAcetylsalicylic acid, nicorandil,	Concurrent coronary and hypertensive left	Intractable diffuse abdominal pain	Nil	Ramipril was discontinued	Recovered

			sive left ventricular dysfunction	escitalopram, and pantoprazole.	ventricular dysfunction				
Augenstein et al. Case 1[6]	Not mentioned, F	Lisinopril	Hypertension			Diffuse abdominal pain, cramping, nausea, and emesis			Recovered
Augenstein et al. Case 2[6]	Not mentioned, F	Lisinopril	Hypertension			Abdominal pain and diarrhoea			Recovered
Neto et al.[7]	43, F	Perindopril 4 mg/day	Hypertension	Nil	Nil	Severe and diffuse abdominal pain	Nil	Perindopril was discontinued, bilastine (10 mg daily for 10 days) and a short prednisolone cycle (20 mg twice/day for 5 days)	Recovered
Razzano et al.[8]	65, M	Lisinopril	Hypertension	Not mentioned	Not mentioned	One episode of " coffee coloured" emesis and nausea	Occasional pain in abdomen for the past few years	Not mentioned.	Not mentioned
Bruetman et al.[9]	74, F	Enalapril 5 mg every 12 hours	Hypertension	Nil	Nil	Severe abdominal pain associated with bloating, vomiting and diarrhoea	Nil	Enalapril was discontinued	Recovered
Siu et al.[10]	58, F	Lisinopril	Not mentioned	Not mentioned	Not mentioned	Acute on chronic non-radiating periumbilical pain with abdominal distension, nausea and emesis	Nil	Lisinopril was discontinued	Recovered
Palmquist et al.[11]	42, F	Lisinopril 5 mg/day	Hypertension	Hydrochlorothiazide, acetaminophen, ibuprofen	Morbid obesity, and obstructive sleep apnoea	Abdominal pain, nausea, and vomiting	Nil	Lisinopril was discontinued, intravenous fluids, pain and nausea	Recovered

								medications.	
Voore et al.[12]	43, F	Lisinopril	Hypertension	Omeprazole and simvastatin	Hyperlipidaemia	Abdominal pain, nausea and vomiting	Nil	Lisinopril was discontinued, intravenous hydration and empiric antibiotics for presumed colitis (Stool- C/S-negative)	Recovered
Syed et al.[13]	60, F	Enalapril	Hypertension	Nil	Nil	Severe abdominal pain, bilious vomitus and watery diarrhoea	Nil	Enalapril was discontinued	Recovered
Oliveira et al. [14]	46, F	Ramipril	Hypertension	Nil	Nil	Crampy abdominal pain, nausea and vomiting	Nil	Ramipril was discontinued	Recovered
Dorsey et al.[15]	45, F	Lisinopril	Hypertension	Levothyroxine and oral contraceptives.	Hypothyroidism	Severe abdominal pain	Seven similar episodes for the past six months	Lisinopril was discontinued	Recovered
Byrne et al. Case 1[16]	67, F	Fosinopril	Hypertension	Acetylsalicylic acidAcetylsalicylic acid, oral estrogen	Right carotid stenosis with one previous transient ischemic attack, undergone hysterectomy and bilateral oophorectomy 30 years previously.	Severe mid-epigastric pain associated with nausea and vomiting	Episodes of tongue swelling and one similar episode before 3 years	Fosinopril was discontinued, intravenous prochlorperazine for nausea, maintenance saline infusion	Recovered
Byrne et al. Case 2[16]	41, F	Lisinopril 20 mg/day	Hypertension	Nil	Nil	Crampy abdominal pain, nausea	One similar episode one month back and undergone cholecystectomy	Lisinopril was discontinued, surgery without resection	Recovered
Yarze et al.[17]	49, F	Lisinopril	Hypertension	Alprazolam, fluoxetine	Anxiety, depression	Acute mid abdominal	Nil	IV hydration,	Recovered

						pain, vomiting and voluminous watery diarrhoea		analgesia and antiemetic therapy	
Johnson et al.[18]	63, F	Lisinopril 30 mg/day	Hypertension	Not mentioned	Hyperlipidaemia, hypothyroidism, diverticulitis	Severe abdominal pain, nausea, diarrhoea	Nil	Lisinopril was discontinued, diphenhydramine, famotidine, and methylprednisolone	Recovered
Gill et al.[19]	49, F	Lisinopril 20 mg/day	Hypertension	Nil	Nil	Abdominal pain, nausea and vomiting	Nil	Lisinopril was discontinued, patient received intravenous hydration and metronidazole.	Recovered
Wuthnow et al. [20]	49, M	Lisinopril	Hypertension	Not mentioned	Type 1 diabetes mellitus	Abdominal pain, nausea, vomiting and diarrhoea	Similar intermittent episodes for the past 5 years.	Lisinopril was discontinued	Recovered
Haines et al.[21]	67, F	Lisinopril 10 mg/day	Hypertension	Hydrochlorothiazide 25 mg daily	Hypertension, dyslipidaemia, and remote (greater than 5 years previous) history of breast cancer	Crampy intermittent abdominal pain, nausea, vomiting, and diarrhoea	Nil	Lisinopril was discontinued, intravenous fluids, NPO status, and nasogastric decompression	Recovered
Gabriel et al.[22]	68, F	Lisinopril 40 mg/day	Hypertension	Nil	Nil	Chronic non-bloody diarrhoea, hypotension and acute kidney injury	Nil	Lisinopril was discontinued	Recovered
Patel et al.[23]	73, M	Benazepril	Hypertension	Nil	Nil	Oropharyngeal edema and gargled speech, colicky abdominal pain	Nil	Benazepril was discontinued, supportive care	Recovered

Velez et al. [24]	52, M	Lisinopril	Hypertension	Acetylsalicylic acid, metoprolol, furosemide, spironolactone, and atorvastatin	Ischemic cardiomyopathy in the setting of hypertension, hyperlipidaemia, type 2 diabetes mellitus, and active tobacco abuse	Abdominal discomfort, diarrhoea, oropharyngeal edema	Nil	Lisinopril was discontinued, prednisone and diphenhydramine	Recovered
Smoger et al. [25]	61, M	Captopril	Hypertension, coronary artery disease, and congestive heart failure	Acetylsalicylic acid, indomethacin, allopurinol, colchicine, and nifedipine	Hypertension, coronary artery disease, and congestive heart failure	Sudden swelling of the lips, face, and tongue, followed by nausea, emesis, abdominal pain, and diarrhoea.	Nil	Captopril was discontinued	Recovered
Aggarwal et al. [26]	45, F	Lisinopril	Hypertension	Nil	Nil	Nausea, vomiting, abdominal pain and diarrhoea	Nil	Lisinopril was discontinued	Recovered
Vallabh et al. [27]	41, F	Lisinopril	Hypertension	Nil	Nil	Abdominal pain and non-bloody diarrhoea.	Similar sporadic episodes for the past two years	Oral prednisone at 60 mg daily showed partial response, bile acid sequestrants, rifaximin, and anticholinergics were trialled unsuccessfully	Recovered
Barnett et al. [28]	43, F	Lisinopril	Hypertension	Adalimumab, metformin, semaglutide, pantoprazole, and oral contraceptive	Diabetes mellitus, hypertension, and psoriasis	Diffuse, contractile abdominal pain with associated nausea, occasional emesis, abdominal distension, subjective fevers and chills	Nil	Lisinopril was discontinued	Recovered

Inayat et al. [29]	53, F	Lisinopril 20 mg/day	Hypertension	Nil	Nil	Abdominal pain and diarrhoea	Nil	Lisinopril was discontinued	Recovered
UY et al. [30]	48, F	Lisinopril	Hypertension	Nil	Organ-limited granulomatosis with polyangiitis	Right lower quadrant abdominal pain	Nil	Lisinopril was discontinued	Recovered
de Graaff et al. [31]	49, F	Lisinopril	Hypertension	Levetiracetam and nitrazepam	Epilepsy and bronchitis	Acute abdominal pain and nausea	Nil	Intestinal ischemia was suspected and an explorative laparotomy abdominal pain despite broad spectrum antibiotics, analgesics, and laxatives. Lisinopril was discontinued	Recovered
Bharwad et al. [32]	49, M	Lisinopril	Hypertension	Nil	Type 1 diabetes mellitus, Grave's disease,	Chronic intermittent abdominal pain, nausea, vomiting, and diarrhoea.	Similar intermittent episodes for the past five years	Lisinopril was discontinued. FODMAP (Fermentable oligosaccharides, disaccharides, monosaccharides and polyols) and lactose-free diets, fiber, cholestyramine, dicyclomine, doxepin, steroids, mesalamine, and antibiotics had been tried without improvement in	Recovered

								symptoms, suggesting irritable bowel syndrome, inflammatory bowel disease, lactose intolerance as unlikely causes. During this time the patient underwent two surgeries: an exploratory laparotomy showing possible evidence of creeping fat but no apparent inflammatory bowel disease, and a cholecystectomy which failed to relieve symptoms.	
Korniyenko et al. [33]	57, F	Lisinopril 20 mg/day	Hypertension	Metformin 500 mg twice a day	Diabetes mellitus	Severe, dull abdominal pain associated with non-bilious vomiting and nausea	Similar episodes of varying severity were seen in the last one year. Exploratory laparotomy failedd to correct the symptoms in one such severe episode.	Lisinopril was discontinued	Recovered
Frutuoso et al. [34]	42, F	Perindopril	Hypertension	Indapamide	Arterial hypertension	Diffuse abdominal pain	Nil	Perindopril was discontinued,	Recovered

								intravenous fluids and analgesics and nil per mouth	
Mullins et al. [35]	59, F	Enalapril	Hypertension			Recurrent abdominal pain, diarrhoea and vomiting	32 similar episodes of abdominal pain in the past 5 years, out of which 12 required analgesics and hospitalization.		Recovered
Gabriel et al. [36]	68, F	Lisinopril	Hypertension	Nil	Nil	Non-bloody diarrhoea occurring for the last one month	Nil	Lisinopril was discontinued	Recovered
Mutnuri et al. [37]	60, F	Enalapril	Hypertension	Hydrochlorothiazide, enalapril, verapamil, and omeprazole	Hypertension and gastroesophageal reflux disease	Abdominal pain and diarrhoea	Similar three episodes for the past two years, which was diagnosed as viral gastroenteritis and treated for the same.	Enalapril was discontinued, supportive care – bowel rest and intravenous hydration.	Recovered
Patel et al. [38]	49, F	Lisinopril 7 years	Hypertension	Nil	Nil	Recurring severe episodes of band-like abdominal pain	Recurrent similar episodes for the past seven years	Lisinopril was discontinued, steroids	Recovered
Pirzada et al. [39]	58, F	Lisinopril 20 mg/day	Hypertension	Chlorthalidone, glimepiride, metformin, oxybutynin, sertraline, trazodone	Diabetes	Abdominal pain, nausea, vomiting	Recurrent similar episodes for the last three years	Lisinopril was discontinued	Recovered
Niyibizi et al.[40]	61, F	Lisinopril 20 mg/day	Hypertension	Albuterol nebulizer, Atorvastatin	Asthma	Severe, diffuse episodic abdominal pain,	Similar episodes in the last two months	Lisinopril was discontinued, NPO, bowel rest,	Recovered

						nausea, cough, vomiting		morphine, acetaminophen	
Wilin et al. [41]	62, F	Lisinopril 40 mg BD	Hypertension	Amlodipine metoprolol tartrate, apixaban, metformin, glipizide, simvastatin ,levetiracetam, ferrous sulfate, ipratropium, calcium carbonate, vitamin D, docusate, pantoprazole, folic acid	Atrial fibrillation, type II diabetes, seizures, iron deficiency anaemia, chronic obstructive pulmonary disease, temporal arteritis, gastroesophageal reflux disease, and hiatal hernia	Nausea abdominal pain	Nil	Lisinopril was discontinued	Recovered
Pinto et al. [42]	31, F	Lisinopril	Hypertension	Nil	Nil	Abdominal pain, lip swelling, nausea, vomiting, diarrhoea, and poor oral intake	Nil	Lisinopril was discontinued, corticosteroids, antihistamines	Recovered
Habib et al. [43]	52, M	Lisinopril	Hypertension	Nil	Nil	Abdominal pain, bloating and watery diarrhoea	Nil	Lisinopril was discontinued	Recovered
Ferreiraa et al. [44]	28, F	Lisinopril	Hypertension	Nil	Nil	Generalized abdominal pain nausea, vomiting, single episode of watery stool	Nil	Lisinopril was discontinued	Recovered
Huynh et al. [45]	44, F	Perindopril 4 mg	Hypertension	Nil	Nil	Intermittent abdominal cramps to severe pain, nausea, vomiting, one episode of non-bloody diarrhoea	Nil	Perindopril was discontinued, analgesics, IV fluids, bowel rest	Recovered
Squillante et al. [46]	40, F	Lisinopril 20 mg 3 days	Hypertension	Nil	Nil	Diffuse abdominal pain, nausea,	Nil	Lisinopril was discontinued, IV fluids,	Recovered

							emesis, and bloating		hydromorphone	
Sravanthi et al. [47]	44, M	Lisinopril		Hypertension	Albuterol inhalation	Asthma	Severe lower abdominal pain	Nil	Lisinopril was discontinued, bowel rest, IV fluids and analgesics	Recovered
Srinivasan et al. [48]	36, F	Lisinopril		Hypertension	Sirolimus, tacrolimus, prednisone	Following transplantation (immunosuppression)	Severe diffuse abdominal pain	Nil	Lisinopril was discontinued, opioids	Recovered
Melendez et al. [49]	53, M	Lisinopril	7 years	Hypertension	Nil	Nil	Severe epigastric pain, nausea, bloating, vomiting	Nil	Lisinopril was discontinued, IV fluids, ondansetron, morphine, NPO	Recovered
Cuypers et al. [50]	38, F	Lisinopril<br>10 mg<br>3 weeks		Dilated cardiomyopathy	Spironolactone, carvedilol	Dilated cardiomyopathy	Lower abdominal pain	Nil	Lisinopril was discontinued, IV fluids	Recovered
Shahani et al. [51]	50, F	Lisinopril		Hypertension	Mesalamine, azathioprine	Crohn’s disease	Severe abdominal pain, nausea and vomiting	Nil	Lisinopril was discontinued, antihistamines	Recovered
Parreira et al. [52]	32, F	Perindopril<br>4 mg<br>1 month		Hypertension	Metoprolol	Hypertension	Diffuse abdominal pain, nausea, bilious vomiting	Nil	Perindopril was discontinued, supportive treatment	Recovered
Ali et al. [53]	46, F	Lisinopril		Hypertension	Nil	Nil	Nausea, vomiting, diarrhoea and abdominal pain	Nil	Lisinopril was discontinued	Recovered
Gurbir et al. [54]	24, F	Lisinopril		Hypertension	Nil	Nil	Abdominal pain, vomiting, and diarrhoea	Nil	Lisinopril was discontinued	Recovered
Dobbels et al.[55]	53, M	Perindopril<br>1 month		Hypertension	Bisoprolol-hydrochlorothiazide, cloxazolam, butyl hyoscine bromide	Hypertension, lactase deficiency, COPD	Abdominal pain, nausea, vomiting	Nil	Perindopril was discontinued, supportive treatment	Recovered

Chawla et al. [56]	34, F	Lisinopril	Hypertension	Nil	Nil	Nausea, abdominal pain	Nil	Lisinopril was discontinued	Recovered
George et al. [57]	43, F	Perindopril 1 month	Hypertension	Nil	Nil	Acute abdominal pain, vomiting and diarrhoea	Nil	Perindopril was discontinued, supportive treatment	Recovered
Goyal et al. [58]	44, F	Lisinopril 2 months	Hypertension	Nil	Nil	Acute onset, diff use abdominal pain, nausea, vomiting, and diarrhoea	Nil	Lisinopril was discontinued, analgesics, NPO	Recovered
Mapakshi et al. [59]	37, F	Lisinopril	Hypertension	Nil	Nil	Generalized post-prandial abdominal pain associated with nausea, vomiting and diarrhoea	Nil	Lisinopril was discontinued	Recovered
Sharma et al. [60]	47, F	Lisinopril 2 years	Hypertension	Nil	Nil	Acute onset, diffuse abdominal pain, nausea and vomiting	Recurrent over the last 2 years	Lisinopril was discontinued	Recovered
Krause et al. [61]	52, F	Lisinopril 5-7 years	Hypertension	Cough syrup, famotidine	Cough	Acute onset of abdominal pain, nausea, emesis and 7 episodes of watery diarrhoea	2 Prior episodes	Lisinopril was discontinued, ceftriaxone, metronidazole, ondansetron, morphine, prednisone	Recovered
Mujer et al. [62]	42, M	Lisinopril	Chronic kidney disease, hypertension	Nil	Nil	Abdominal Pain nausea, intractable vomiting, dyspnoea and dizziness	Nil	Lisinopril was discontinued	Recovered
Vilar et al. [63]	48, M	Enalapril	Hypertension	Metformin, simvastatin	Diabetic dyslipidemia	Sudden onset of colicky pain in the umbilicus, dizziness,	Nil	Enalapril was discontinued	Recovered

						general malaise, without nausea, vomiting or diarrhoea.			
Mir et al. [64]	23, F	Lisinopril	Hypertension	Nil	Nil	Abdominal pain	Nil	Lisinopril was discontinued	Recovered
Adusumilli et al. [65]	34, F	Lisinopril	Hypertension	Nil	Nil	Nausea, vomiting, epigastric pain	Nil	Lisinopril was discontinued	Recovered
Shahzad et al. [66]	45, F	Benazepril 5 mg	Hypertension	Amlodipine	Hypertension	Abdominal pain, nausea and vomiting	Nil	Benazepril was discontinued	Recovered
Scott et al.[67]	61, M	Benazepril 20 mg 2 years	Hypertension	Nil	Nil	Intermittent abdominal pain and bloating	Nil	Benazepril was discontinued	Recovered
Bloom et al. [68]	51, M	Captopril 9 years	Hypertension	Metoprolol, Acetylsalicylic acid	Hypertension	Generalized abdominal pain	Nil	Captopril was discontinued, supportive treatment	Recovered
Arshad et al. [69]	85, F	Lisinopril	Hypertension	Nil	Nil	Abdominal pain, nausea, vomiting, small bowel obstruction	Nil	Lisinopril was discontinued, IV fluids, nasogastric suction, antihistamines, steroids, fresh frozen plasma	Recovered
Rosenberg et al. [70]	38, F	Enalapril 2.5 mg 2 days	Hypertension (related to calcineurin inhibitors)	Azathioprine, Cyclosporin	Post liver transplant, long-term maintenance immunosuppression regimen	Abdominal pain, nausea, vomiting	Nil	Enalapril was discontinued, IV fluids, nil per oral, antibiotics	Recovered
Mingos et al [71]	34, F	Angiotensin-converting enzyme inhibitor	Background of renal transplant for medullary cystic kidney disease	Nil	Nil	12 years of stereotyped episodes of abdominal pain with loose stools	Recurrent episodes for 12 years	Angiotensin-converting enzyme inhibitor was discontinued	Recovered

Marmery et al.[72]	48, F	Lisinopril 5 mg	Proteinuria	Nil	Nil	Diarrhoea and vomiting	Nil	Lisinopril was discontinued	Recovered
Tsuboi et al. [73]	42, M	Lisinopril 2 days	Following cerebral infarction	Nil	Nil	Acute abdominal pain	Nil	Lisinopril was discontinued	Not mentioned
Weingärtner et al. [74]	67, F	Ramipril 5 mg	Hypertensive cardiomyopathy	Metoprolol	Hypertension	Hoarse, raspy voice, progressive abdominal pain, difficulty breathing	Nil	Ramipril was discontinued, epinephrine, antihistamines, corticosteroids.	Recovered
Khan et al. [75]	42, F	Benazepril	Hypertension	Amlodipine	Hypertension	Dull ache in the center of the abdomen, non-projectile bilious vomiting	Nil	Benazepril was discontinued	Recovered
Burroughs et al.[76]	75, F	Lisinopril	Hypertension	Nil	Nil	Recurrent episodes of abdominal pain and watery diarrhoea	Nil	Lisinopril was discontinued	Recovered
Spahn et al. [77]	40, F	Enalapril 5 mg 10 weeks	Hypertension	Bisoprolol	Hypertension	Relapsing abdominal pain, nausea and vomiting	Nil	Enalapril was discontinued	Recovered
Rashad et al. [78]	41, F	Lisinopril	Hypertension	Nil	Nil	Recurrent episodes of periumbilical and epigastric abdominal pain, nausea, vomiting, and watery diarrhoea	Nil	Lisinopril was discontinued	Recovered
Zeng et al. [79]	23, F	Enalapril 2 months	Not mentioned	Nil	Nil	Acute abdominal pain	Nil	Lisinopril was discontinued, supportive treatment	Recovered

Jani et al. [80]	58, M	Lisinopril	Hypertension	Acetylsalicylic acid, simvastatin, sumatriptan	Not mentioned	Acute onset generalized abdominal pain, non-bloody non-bilious vomiting	Nil	Lisinopril was discontinued	Recovered
Antulov et al. [81]	45, F	Enalapril 6 months	Hypertension	Nil	Nil	Diffuse abdominal pain, nausea, heartburn, and vomiting	Nil	Lisinopril was discontinued	Recovered
Arakawa et al. [82]	44, F	Lisinopril 10 mg Valsartan 80mg 1 month	Hypertension	Nil	Nil	Acute onset of abdominal pain	Nil	Lisinopril was discontinued	Recovered
Wojciechowska et al. [83]	33, M	Perindopril	Hypertension	Nil	Nil	Lower abdominal pain, urge to have bowel movement, and difficulty passing stool	Nil	Perindopril was discontinued, antihistamines, analgesics, intravenous fluids	Recovered

HAE, hereditary angioedema; C1 INH, C1 inhibitor; BK, bradykinin; ACE-I, angiotensin converting enzyme inhibitor; CPN, carboxypeptidase N; AA, arachidonic acid; NO, nitric oxide; PG, prostaglandins; APP, aminopeptidase P; DPP, dipeptidyl peptidase.

References

1. Myslinski, J.; Heiser, A.; Kinney, A. Hypovolemic Shock Caused by Angiotensin-Converting Enzyme Inhibitor-Induced Visceral Angioedema: A Case Series and A Simple Method to Diagnose This Complication in the Emergency Department. *J Emerg Med* **2018**, *54*, 375–379, doi:10.1016/j.jemermed.2017.12.009.
2. Orr, K.K.; Myers, J.R. Intermittent Visceral Edema Induced by Long-Term Enalapril Administration. *Ann Pharmacother* **2004**, *38*, 825–827, doi:10.1345/aph.1D458.
3. Salloum, H.; Locher, C.; Chenard, A.; Bigorie, B.; Beroud, P.; Gatineau-Sailliant, G.; Glikmanas, M. [Small bowel angioedema due to perindopril]. *Gastroenterol Clin Biol* **2005**, *29*, 1180–1181, doi:10.1016/s0399-8320(05)82187-6.
4. Gillion, V.; Dragean, C.A.; Dahlqvist, G.; Jadoul, M. Intestinal Angioedema from Angiotensin Converting Enzyme Inhibitor. *Kidney Int* **2019**, *96*, 798, doi:10.1016/j.kint.2019.02.031.
5. Dietler, V.; Fusi-Schmidhauser, T. Intestinal Angioedema in a Palliative Care Setting. *Am J Med* **2016**, *129*, e293–e294, doi:10.1016/j.amjmed.2016.06.053.
6. Augenstein, V.A.; Heniford, B.T.; Sing, R.F. Intestinal Angioedema Induced by Angiotensin-Converting Enzyme Inhibitors: An Underrecognized Cause of Abdominal Pain? *J Am Osteopath Assoc* **2013**, *113*, 221–223.
7. Ecim 2019, A. of Abstract Book of the 18th Conference in Internal Medicine. *European Journal of Case Reports in Internal Medicine* **2019**, doi:10.12890/2019_V6Sup1.
8. Razzano, A.; Alagheband, S.; Ahmed, H.; Malet, P.; Katz, D. Isolated Angiotensin Converting Enzyme (ACE) Inhibitor Induced Small Bowel Angioedema After 10 Years of Oral Lisinopril Therapy: 2211. *Official journal of the American College of Gastroenterology | ACG* **2016**, *111*, S1060.

9. Bruetman, J.E.; Montes Onganía, A.; Finn, B.C.; Young, P. [Isolated intestinal angioedema induced by enalapril]. *Medicina (B Aires)* **2018**, *78*, 41–43.
10. Siu, L.; Ahmed, A.; Grossman, E. Isolated Intestinal Involvement of Angioedema Induced by ACE Inhibitor: 332. *Official journal of the American College of Gastroenterology | ACG* **2013**, *108*, S100.
11. Palmquist, S.; Mathews, B. Isolated Intestinal Type Angioedema Due to ACE-Inhibitor Therapy. *Clin Case Rep* **2017**, *5*, 707–710, doi:10.1002/ccr3.925.
12. Voore, N.; Stravino, V. Isolated Small Bowel Angio-Oedema Due to ACE Inhibitor Therapy. *BMJ Case Rep* **2015**, *2015*, bcr2015212623, doi:10.1136/bcr-2015-212623.
13. Syed, M.; Rey-Mendoza, J.; Simons-Linares, R.C.; Stroger, J.H. Isolated Small Intestine Angioedema: An Under-Recognized Complication of Angiotensin-Converting Enzyme Inhibitor Therapy: 2504. *Official journal of the American College of Gastroenterology | ACG* **2017**, *112*, S1367.
14. Oliveira, A.M.; Santiago, I.; Carvalho, R.; Martins, A.; Reis, J. Isolated Visceral Angioedema Induced by Angiotensin-Converting Enzyme Inhibitor. *GE Port J Gastroenterol* **2016**, *23*, 162–165, doi:10.1016/j.jpge.2015.09.008.
15. Dorsey, T.J.; Tran, L. Isolated Visceral Angioedema: An Uncommon Complication of ACEI Therapy: 2495. *Official journal of the American College of Gastroenterology | ACG* **2017**, *112*, S1362.
16. Byrne, T.J.; Douglas, D.D.; Landis, M.E.; Heppell, J.P. Isolated Visceral Angioedema: An Underdiagnosed Complication of ACE Inhibitors? *Mayo Clin Proc* **2000**, *75*, 1201–1204, doi:10.4065/75.11.1201.
17. Yarze, J.C.; Sablich, D. Lisinopril-Induced Small Bowel Angioedema: 2511. *Official journal of the American College of Gastroenterology | ACG* **2017**, *112*, S1370.
18. Johnson, B.W.; Rydburg, A.M.; Do, V.D. Lisinopril-Induced Small Bowel Angioedema: An Unusual Cause of Severe Abdominal Pain. *Am J Case Rep* **2022**, *23*, e937895, doi:10.12659/AJCR.937895.
19. Gill, I.; Shaheen, A.A.; Edhi, A.I.; Cappell, M.S. S2882 Mesenteric Angioedema Induced by... : Official Journal of the American College of Gastroenterology | ACG. **2025**.
20. Wuthnow, C.; Bharwad, A.; Rowe, K. S3458 Often Reported, Rarely Considered: A Case of ACEI-Induced Mesenteric Angioedema. *Official journal of the American College of Gastroenterology | ACG* **2022**, *117*, e2179, doi:10.14309/01.ajg.0000870472.17012.95.
21. Haines, E.C.; Wall, G.C. Possible Angiotensin-Converting Enzyme Inhibitor (ACEI)-Induced Small Bowel Angioedema. *J Pharm Pract* **2011**, *24*, 564–567, doi:10.1177/0897190011406126.
22. Gabriel, J.G.; Vedantam, V.; Kapila, A.; Bajaj, K. Recognizing a Rare Phenomenon of Angiotensin-Converting Enzyme Inhibitors: Visceral Angioedema Presenting with Chronic Diarrhea-A Case Report. *Perm J* **2018**, *22*, 17–030, doi:10.7812/TPP/17-030.
23. Patel, Y.; Sekhon, N.; Sharma, N.; Ramakrishna, S.; Ochieng, P. 587: RECURRENT BOWEL ANGIOEDEMA DIAGNOSED RETROSPECTIVELY AFTER OROPHARYNGEAL ANGIOEDEMA. *Critical Care Medicine* **2022**, *50*, 285, doi:10.1097/01.ccm.0000808672.57442.0d.
24. Abstracts from the 37th Annual Meeting of the Society of General Internal Medicine | Journal of General Internal Medicine Available online: https://link.springer.com/article/10.1007/s11606-014-2834-9 (accessed on 10 March 2025).
25. Smoger, S.H.; Sayed, M.A. Simultaneous Mucosal and Small Bowel Angioedema Due to Captopril. *South Med J* **1998**, *91*, 1060–1063, doi:10.1097/00007611-199811000-00015.
26. Aggarwal, A.; Mehta, N.; Shah, S.N. Small Bowel Angioedema Associated with Angiotensin Converting Enzyme Inhibitor Use. *J Gen Intern Med* **2011**, *26*, 446–447, doi:10.1007/s11606-010-1559-7.
27. Vallabh, H.; Hahn, B.; Bryan, C.; Hogg, J.; Kupec, J.T. Small Bowel Angioedema from Angiotensin-Converting Enzyme: Changes on Computed Tomography. *Radiol Case Rep* **2018**, *13*, 55–57, doi:10.1016/j.radcr.2017.09.014.
28. Barnett, J.; Yu, K.K.; Mayilvaganan, B. S4714 Small Bowel Angioedema Secondary to ACE Inhibitor. *Official journal of the American College of Gastroenterology | ACG* **2024**, *119*, S2983, doi:10.14309/01.ajg.0001048224.41507.6c.

29. Inayat, F.; Hurairah, A. Small Bowel Angioedema Secondary to Angiotensin-Converting Enzyme Inhibitors. *Cureus* **2016**, *8*, e943, doi:10.7759/cureus.943.
30. Uy, P.P.; Yap, J.E. 2601 Small Intestine Angioedema Due to Angiotensin Converting Enzyme Inhibitors: A Great Mimicker. *Official journal of the American College of Gastroenterology | ACG* **2019**, *114*, S1432, doi:10.14309/01.ajg.0000599936.20541.a6.
31. de Graaff, L.C.G.; van Essen, M.; Schipper, E.M.; Boom, H.; Duschek, E.J.J. Unnecessary Surgery for Acute Abdomen Secondary to Angiotensin-Converting Enzyme Inhibitor Use. *Am J Emerg Med* **2012**, *30*, 1607–1612, doi:10.1016/j.ajem.2011.10.028.
32. Bharwad, A.; Wuthnow, C.; Mahdi, M.; Rowe, K. Unresolved Chronic Diarrhea: A Case of Angiotensin-Converting Enzyme Inhibitor-Induced Mesenteric Angioedema. *Eur J Case Rep Intern Med* **2023**, *10*, 003995, doi:10.12890/2023_003995.
33. Korniyenko, A.; Alviar, C.L.; Cordova, J.P.; Messerli, F.H. Visceral Angioedema Due to Angiotensin-Converting Enzyme Inhibitor Therapy. *Cleve Clin J Med* **2011**, *78*, 297–304, doi:10.3949/ccjm.78a.10102.
34. Frutuoso, B.; Esteves, J.; Silva, M.; Gil, P.; Carneiro, A.C.; Vale, S. Visceral Angioedema Induced by Angiotensin Converting Enzyme Inhibitor: Case Report. *GE Port J Gastroenterol* **2016**, *23*, 166–169, doi:10.1016/j.jpge.2015.10.004.
35. Mullins, R.J.; Shanahan, T.M.; Dobson, R.T. Visceral Angioedema Related to Treatment with an ACE Inhibitor. *Med J Aust* **1996**, *165*, 319–321, doi:10.5694/j.1326-5377.1996.tb124991.x.
36. Gabriel, J.; Balagoni, H.; Kapila, A.; Bajaj, K. Visceral Angioedema: A Rare Complication of ACE Inhibitors Causing Chronic Diarrhea. *American Journal of Gastroenterology* **2016**, *111*, S990.
37. Mutnuri, S.; Khan, A.; Variyam, E.P. Visceral Angioedema: An under-Recognized Complication of Angiotensin-Converting Enzyme Inhibitors. *Postgrad Med* **2015**, *127*, 215–217, doi:10.1080/00325481.2015.1001305.
38. A Case Isolated Intestinal Angioedema - Record Details - Embase Available online: https://www.embase.com/records?subaction=viewrecord&rid=9&page=1&id=L636473581 (accessed on 24 February 2025).
39. Pirzada, S.; Raza, B.; Mankani, A.A.; Naveed, B. A Case of Angiotensin-Converting Enzyme (ACE) Inhibitor-Induced Small Bowel Angioedema. *Cureus* **2023**, *15*, e47739, doi:10.7759/cureus.47739.
40. Niyibizi, A.; Cisse, M.S.; Rovito, P.F.; Puente, M. Angiotensin-Converting Enzyme (ACE) Inhibitor-Induced Angioedema of the Small Bowel: A Diagnostic Dilemma. *J Am Board Fam Med* **2023**, *36*, 160–163, doi:10.3122/jabfm.2022.220276R1.
41. Wilin, K.L.; Czupryn, M.J.; Mui, R.; Renno, A.; Murphy, J.A. ACE Inhibitor-Induced Angioedema of the Small Bowel: A Case Report and Review of the Literature. *J Pharm Pract* **2018**, *31*, 99–103, doi:10.1177/0897190017690641.
42. Mb, P.; M, V.; Ma, S. A Gut Feeling: Isolated Small Bowel Angioedema Due to Angiotensin-Converting Enzyme Inhibitor. *Rhode Island medical journal (2013)* **2022**, *105*.
43. A Rare Case of ACE Inhibitor-Induced Intestinal Angioedema Presenting as a Delayed Complication in a Heart Transplant Recipient - Record Details - Embase Available online: https://www.embase.com/records?subaction=viewrecord&rid=1&page=1&id=L2017590450 (accessed on 24 February 2025).
44. Ferreira, T.A.; Alves, M.R.; Oliveira, A.M.P.; Silva, F.S.S.; Pereira, C. A Rare Cause of Abdominal Pain: Intestinal Angioedema. *J Med Cases* **2021**, *12*, 138–140, doi:10.14740/jmc3651.
45. Huynh, T.N.A.; Hua, L.; Smalberger, J.A.; James, J. ACE Inhibitor Induced Intestinal Angioedema. *ANZ J Surg* **2022**, *92*, 3110–3111, doi:10.1111/ans.17575.
46. Squillante, M.D.; Trujillo, A.; Norton, J.; Bansal, S.; Dragoo, D. ACE Inhibitor Induced Isolated Angioedema of the Small Bowel: A Rare Complication of a Common Medication. *Case Rep Emerg Med* **2021**, *2021*, 8853755, doi:10.1155/2021/8853755.
47. Sravanthi, M.V.; Suma Kumaran, S.; Sharma, N.; Milekic, B. ACE Inhibitor Induced Visceral Angioedema: An Elusive Diagnosis. *BMJ Case Rep* **2020**, *13*, e236391, doi:10.1136/bcr-2020-236391.

48. Srinivasan, D.; Strohbehn, G.W.; Cascino, T. ACE Inhibitor-Associated Intestinal Angioedema in Orthotopic Heart Transplantation. *ESC Heart Fail* **2017**, *4*, 384–386, doi:10.1002/ehf2.12161.
49. Melendez, M.; Grosel, J.M. ACE Inhibitor-Induced Angioedema Causing Small Bowel Obstruction. *JAAPA* **2020**, *33*, 28–31, doi:10.1097/01.JAA.0000668864.61980.c0.
50. Cuypers, S.; Van Meerbeeck, S.; De Pauw, M. ACE Inhibitor-Induced Angioedema of the Small Intestine: A Case Report. *Acta Cardiol* **2011**, *66*, 645–648, doi:10.1080/ac.66.5.2131092.
51. Shahani, L. ACE Inhibitor-Induced Intestinal Angio-Oedema: Rare Adverse Effect of a Common Drug. *BMJ Case Rep* **2013**, *2013*, bcr2013200171, doi:10.1136/bcr-2013-200171.
52. ACE Inhibitor-Induced Small Bowel Angioedema, Mimicking an Acute Abdomen - PubMed Available online: https://pubmed.ncbi.nlm.nih.gov/33072254/ (accessed on 24 February 2025).
53. Ace Inhibitor-Induced Visceral Angioedema - Record Details - Embase Available online: https://www.embase.com/records?subaction=viewrecord&rid=10&page=1&id=L71750334 (accessed on 24 February 2025).
54. Ace Inhibitor-Induced Visceral Angioedema-a Rare Phenomenon - Record Details - Embase Available online: https://www.embase.com/records?subaction=viewrecord&rid=1&page=1&id=L630841473 (accessed on 24 February 2025).
55. Dobbels, P.; Van Overbeke, L.; Vanbeckevoort, D.; Hiele, M. Acute Abdomen Due to Intestinal Angioedema Induced by ACE Inhibitors: Not so Rare? *Acta Gastroenterol Belg* **2009**, *72*, 455–457.
56. Acute Abdomen Due to Large Bowel Angioedema Caused by Angiotensin Converting Enzyme Inhibitor - Record Details - Embase Available online: https://www.embase.com/records?subaction=viewrecord&rid=1&page=1&id=L70698275 (accessed on 24 February 2025).
57. Acute Abdominal Pain in a Patient Taking ACE-I - Record Details - Embase Available online: https://www.embase.com/records?subaction=viewrecord&rid=1&page=1&id=L71279815 (accessed on 24 February 2025).
58. Alimentary, My Dear Watson! ACE Inhibitor-Induced Bowel Angioedema - Record Details - Embase Available online: https://www.embase.com/records?subaction=viewrecord&rid=1&page=1&id=L71749985 (accessed on 24 February 2025).
59. An Ace Inhibitor of Spades: An Unsual Cause of Enteritis - Record Details - Embase Available online: https://www.embase.com/records?subaction=viewrecord&rid=1&page=1&id=L71878150 (accessed on 24 February 2025).
60. An Unusual Case of Recurrent Abdominal Pain: Ace Inhibitor Induced Visceral Angioedema - Record Details - Embase Available online: https://www.embase.com/records?subaction=viewrecord&rid=1&page=1&id=L636474361 (accessed on 24 February 2025).
61. Krause, A.J.; Patel, N.B.; Morgan, J. An Unusual Presentation of ACE Inhibitor-Induced Visceral Angioedema. *BMJ Case Rep* **2019**, *12*, e230865, doi:10.1136/bcr-2019-230865.
62. Mujer, M.T.P.; Rai, M.P.; Nemakayala, D.R.; Yam, J.L. Angioedema of the Small Bowel Caused by Lisinopril. *Drug Ther Bull* **2019**, *57*, 14–15, doi:10.1136/dtb.2018.225396rep.
63. Romeu Vilar, D.; López Rey, D. [Angioedema of the small bowel secondary to treatment with angiotensin converting enzyme inhibitor]. *Radiologia* **2015**, *57*, 449–450, doi:10.1016/j.rx.2015.01.005.
64. Mir, A.S.; Sorrentino, D. Angiotensin Converting Enzyme Inhibitor Induced Small Bowel Angioedema. *Dig Liver Dis* **2021**, *53*, 1661, doi:10.1016/j.dld.2020.08.032.
65. Adusumilli, R.K.; Patel, M.; Hong, G.; Kulairi, Z.I. 2631 Angiotensin Converting Enzyme Inhibitor-Induced Isolated Angioedema of Small Bowel. *Official journal of the American College of Gastroenterology | ACG* **2019**, *114*, S1448, doi:10.14309/01.ajg.0000600056.94098.5d.

66. Shahzad, G.; Korsten, M.A.; Blatt, C.; Motwani, P. Angiotensin-Converting Enzyme (ACE) Inhibitor-Associated Angioedema of the Stomach and Small Intestine: A Case Report. *Mt Sinai J Med* **2006**, *73*, 1123–1125.
67. ABSTRACTS PRESENTED AT POSTER SESSIONS NOVEMBER 7-8, 2009 MIAMI BEACH CONVENTION CENTER. *Annals of Allergy, Asthma & Immunology* **2009**, *103*, A93–A146, doi:10.1016/S1081-1206(10)60678-3.
68. Bloom, A.S.; Schranz, C. Angiotensin-Converting Enzyme Inhibitor-Induced Angioedema of the Small Bowel-A Surgical Abdomen Mimic. *J Emerg Med* **2015**, *48*, e127-129, doi:10.1016/j.jemermed.2015.01.016.
69. ANGIOTENSIN-CONVERTING ENZYME INHIBITOR-INDUCED INTESTINAL ANGIOEDEMA: A RARE SIDE EFFECT OF A COMMON DRUG - Record Details - Embase Available online: https://www.embase.com/records?subaction=viewrecord&rid=1&page=1&id=L2027228533 (accessed on 24 February 2025).
70. Rosenberg, E.I.; Mishra, G.; Abdelmalek, M.F. Angiotensin-Converting Enzyme Inhibitor-Induced Isolated Visceral Angioedema in a Liver Transplant Recipient. *Transplantation* **2003**, *75*, 730–732, doi:10.1097/01.TP.0000048491.67462.DA.
71. Mingos, N.; Tjandra, D.; Lim, B.; Hebbard, G. Angiotensin-Converting Enzyme Inhibitor-Induced Small Bowel Angioedema: An Important Differential for Episodic Enteritis. *ACG Case Rep J* **2022**, *9*, e00877, doi:10.14309/crj.0000000000000877.
72. Marmery, H.; Mirvis, S.E. Angiotensin-Converting Enzyme Inhibitor-Induced Visceral Angioedema. *Clin Radiol* **2006**, *61*, 979–982, doi:10.1016/j.crad.2006.06.010.
73. Tsuboi, M.; Sada, R. Angiotensin-Converting-Enzyme Inhibitor-Induced Intestinal Angioedema. *Intern Med* **2015**, *54*, 3247, doi:10.2169/internalmedicine.54.5453.
74. Weingärtner, O.; Weingärtner, N.; Böhm, M.; Laufs, U. Bad Gut Feeling: ACE Inhibitor Induced Intestinal Angioedema. *BMJ Case Rep* **2009**, *2009*, bcr09.2008.0868, doi:10.1136/bcr.09.2008.0868.
75. Khan, M.U.; Baig, M.A.; Javed, R.A.; Ali, S.; Qamar, U.R.; Vasavada, B.C.; Khan, I.A. Benazepril Induced Isolated Visceral Angioedema: A Rare and under Diagnosed Adverse Effect of Angiotensin Converting Enzyme Inhibitors. *Int J Cardiol* **2007**, *118*, e68-69, doi:10.1016/j.ijcard.2007.01.011.
76. Burroughs, S.; Benjamin, A.; Khattab, A.; Fine, M. S3021 Development of Isolated Intestinal Angioedema 10 Years After Initiation of Lisinopril. *Official journal of the American College of Gastroenterology | ACG* **2021**, *116*, S1249, doi:10.14309/01.ajg.0000785616.01197.4f.
77. Spahn, T.W.; Grosse-Thie, W.; Mueller, M.K. Endoscopic Visualization of Angiotensin-Converting Enzyme Inhibitor-Induced Small Bowel Angioedema as a Cause of Relapsing Abdominal Pain Using Double-Balloon Enteroscopy. *Dig Dis Sci* **2008**, *53*, 1257–1260, doi:10.1007/s10620-007-9877-2.
78. Gastroenterologist’s Recognition of Rare Adverse Drug Effect Prevents Further Harm in a Patient with Small Bowel Obstruction Due to Lisinopril: A Case Report and Literature Review - Record Details - Embase Available online: https://www.embase.com/records?subaction=viewrecord&rid=1&page=1&id=L646034839 (accessed on 24 February 2025).
79. Zeng, G.; Li, Y. Gastrointestinal: Small Intestinal Angioedema Induced by Angiotensin-Converting Enzyme Inhibitors. *J Gastroenterol Hepatol* **2024**, *39*, 1967–1968, doi:10.1111/jgh.16583.
80. (PDF) Lisinopril Induced Visceral Angioedema. *ResearchGate* **2024**, doi:10.22158/rhs.v6n3p16.
81. Rincic Antulov, M.; Båtevik, R.B. Angiotensin-Converting Enzyme Inhibitor-Induced Gastrointestinal Angioedema: The First Danish Case Report. *Case Rep Gastroenterol* **2018**, *12*, 556–558, doi:10.1159/000486952.
82. Arakawa, M.; Murata, Y.; Rikimaru, Y.; Sasaki, Y. Drug-Induced Isolated Visceral Angioneurotic Edema. *Intern Med* **2005**, *44*, 975–978, doi:10.2169/internalmedicine.44.975.

83. Wojciechowska, E.; Gryglas, P.; Dul, P. GASTROINTESTINAL ANGIONEUROTIC EDEMA AS A CONSEQUENCE OF ANGIOTENSIN – CONVERTING ENZYME INHIBITOR TREATMENT. *Journal of Hypertension* **2023**, *41*, e312, doi:10.1097/01.hjh.0000942256.38394.9a.
