# Supplementary material for: Drug-Induced Intestinal Angioedema: A Disproportionality Analysis Using the United States Food and Drug Administration Adverse Event Reporting System Database and Literature Review
Source: Med Sci (Basel). 2025 Dec 18;13(4):327. doi: 10.3390/medsci13040327 (PMC12735144; doi:10.3390/medsci13040327)
Supplement: Supplementary file 1 [file medsci-13-00327-s001.zip › Supplementary Table S2.pdf]

Supplementary Table S2. Case reports of intestinal angioedema induced by drugs other than angiotensin converting enzyme inhibitors.

Author	Age (in Years) and Gender	Suspect Drug and Regimen	Indication(s)	Concomitant Medication(s)	Concomitant Medication Indication(s)	Symptoms	Past History of Angioedema	ADR Management	Outcome
Malcolm et al. [1]	58, F	Hormone replacement therapy	Hysterectomy for menorrhagia	Prednisone	Not mentioned	Recurrent crampy abdominal pain, nausea and vomiting	Four similar episodes in the past 3 years	Danazol, and hormone replacement therapy was discontinued	Recovered
Hu et al. [2] Case 1	55, M	90 mL nonionic iodinated contrast media (370 mg iodine/mL) was administered intravenously at a rate of 3 mL/s	Upper abdominal multi-phase contrast-enhanced CT examination	400 mL of iso-osmotic mannitol solution 30 min before the study and 100 mL just before the upper abdominal multi-phase	Upper abdominal multi-phase contrast-enhanced CT examination	Mild abdominal discomfort and diarrhoea	Nil	Resolved spontaneously	Recovered
Hu et al. [2] Case 2	46, M	Iopamidol injection (370 mg iodine/mL)	Complete abdominal contrast-enhanced CT exam	1000 mL oral iso-osmotic mannitol solution	Multiple colon polyps-complete abdominal contrast-enhanced CT exam	Mild abdominal discomfort	Nil	Resolved spontaneously	Recovered
Hu et al. [2] Case 3	32, F	Iohexol injection (350 mg iodine/mL)	Multi-phase abdominal enhanced CT exam	400 mL of iso-osmotic mannitol solution 30 min before the study and 100 mL just before the upper abdominal multi-phase	A mass in the right kidney, referred for a multi-phase abdominal enhanced CT exam	Mild abdominal discomfort	Nil	Resolved spontaneously	Recovered
Osman et al. [3]	80, F	Aspirin 325 mg BD	Lumbago	Lisinopril, tacrolimus and prednisone	Hypertension, end-stage renal disease secondary to ANCA-associated vasculitis, status post kidney transplant	Acute epigastric pain, nausea, and vomiting	Similar multiple episodes over last one year	Bowel rest and intravenous hydration, lisinopril was switched to amlodipine, acetylsalicylic acid was discontinued	Recovered
Turcu et al. [4]	56, F	Calcium channel blocker	Not mentioned	Nil	Nil	Recurrent diffuse, intermittent abdominal pain, nausea,	Eight similar episodes in last two years	Calcium channel blocker was discontinued	Recovered

						vomiting, and diarrhoea			
Alawami et al[5]	47, F	Hyaluronic acid–based dermal fillers	Not mentioned	Irbesartan	Hypertension	Episodic abdominal pain	Similar symptoms for the past 3 months. She experienced recurrent facial and lip swelling	Hyaluronidase was injected	Recovered
Mousa et al [6]	51, M	Losartan 50 mg/day	Hypertension	40 mg of pantoprazole	Reflux esophagitis	Episodic diarrhoea with abdominal pain	3-year history of nontender, non-itchy swelling of his upper and lower extremities and scrotum. Each episode lasted for 5 to 8 weeks prior to resolution. He was on enalapril for the hypertension but was given losartan 3 years prior because he developed a drug-induced cough.	Losartan was discontinued	Recovered
Majoni et al [7]	43, M	Losartan	Hypertension	Nil	Hereditary angioedema, nephrotic syndrome	Worsening nephrotic syndrome and abdominal pain	Nil	Losartan was discontinued. Infusion of C1 INH concentrate, increased doses of prednisolone and furosemide, cyclosporine was added	Recovered
Yang et al [8]	38, F	Sirolimus 1mg orally QD for maintenance therapy	Renal transplantation	Thymoglobulin for induction immunosuppressive therapy, and tacrolimus,	Renal transplantation	Generalized gastric pain, nausea, vomiting, and diarrhoea	Nil	Sirolimus was discontinued	Recovered

				glucocorticoids for maintenance therapy					
Maarek et al [9]	34, M	10 ml gadobenate dimeglumine	Contrast agents for imaging	Nil	Nil	Abdominal cramps	Nil	Resolved spontaneously	Recovered
Park et al [10] Case 1	46, M	150 ml nonionic contrast medium	Abdominopelvic CT	300 mL barium sulfate suspension oral contrast medium and additional 150 mL just before scanning	Abdominopelvic CT	Vomiting, sneezing, and nasal obstruction	Nil	Hydration and antihistamine	Recovered
Park et al. [10] Case 2	49, M	150 ml nonionic contrast medium	Abdominopelvic CT	300 mL barium sulfate suspension oral contrast medium and additional 150 mL just before scanning	Abdominopelvic CT	Gastrointestinal symptoms with vomiting	Nil	Hydration and medical observation	Recovered
Park et al. [10] Case 3	57, M	150 ml nonionic contrast medium	Abdominopelvic CT	300 mL barium sulfate suspension oral contrast medium and additional 150 mL just before scanning	Abdominopelvic CT	Gastrointestinal symptoms with vomiting	Nil	Resolved spontaneously	Recovered
Park et al. [10] Case 4	48, M	150 ml nonionic contrast medium	Abdominopelvic CT	Nil	Abdominopelvic CT	Gastrointestinal symptoms with vomiting	Nil	Hydration treatment plus medical observation	Recovered
Zvidi et al. [11]	19, F	Tacrolimus	Orthotopic kidney transplantation	Prednisone, lercanidipine, enalapril, prolol, clonidine, omeprazole, furosemide and calcium	Orthotopic kidney transplantation	Severe, watery diarrhoea, diffuse abdominal pain and fatigue	Nil	Tacrolimus and ACE-I were discontinued	Recovered
Steven et al. [12]	81, F	Losartan	Hypertension	Nil	Nil	Abdominal pain, nausea, single episode of bloody diarrhoea	Nil	Resolved spontaneously	Recovered
Jordan Orr et al. [13]	59, F	Irbesartan	Not mentioned	Nil	Nil	Severe cramping periumbilical abdominal pain	Nil	Resolved spontaneously	Recovered
Thalanayar et al. [14]	34, F	Losartan	Hypertension with end stage renal disease	Nifedipine, clonidine	Hypertension	Severe abdominal pain, nausea vomiting, and diarrhoea	Similar past episodes	Resolved spontaneously	Recovered

Ghezzi et al. [15]	82, F	Losartan 50 mg/day 4 months	Hypertension	Nil	Nil	Mid-epigastric pain, vomiting and diarrhoea	Nil	Resolved spontaneously	Recovered
Kim et al. [16] Case 1	51, M	Diatrizoate meglumine and diatrizoate sodium solution intravenous, iopromide	Contrast-enhanced abdominal CT	Nil	Nil	Mild abdominal discomfort	Nil	Resolved spontaneously	Recovered
Kim et al. [16] Case 2	40, M	Diatrizoate meglumine and diatrizoate sodium solution intravenous, iopromide	Contrast-enhanced abdominal CT	Nil	Nil	Mild abdominal discomfort	Nil	Resolved spontaneously	Recovered
Kim et al. [16] Case 3	48, M	Diatrizoate meglumine and diatrizoate sodium solution intravenous, iopromide	Contrast-enhanced abdominal CT	Nil	Nil	Mild abdominal discomfort	Nil	Resolved spontaneously	Recovered
Chen et al. [17]	75, M	Iopamidol 80 mL	Preoperative staging abdominal computed tomography	Nil	Nil	Mild abdominal fullness	Nil	Resolved spontaneously	Recovered

HAE, hereditary angioedema, CT, computed tomography; COX-1, cyclooxygenase 1; AA, arachidonic acid; BK, bradykinin.

References

1. Malcolm, A.; Prather, C.M. Intestinal Angioedema Mimicking Crohn's Disease. *Med J Aust* **1999**, *171*, 418–420, doi:10.5694/j.1326-5377.1999.tb123722.x.
2. Hu, X.-H.; Gong, X.-Y.; Hu, P. Transient Small Bowel Angioedema Due to Intravenous Iodinated Contrast Media. *World J Gastroenterol* **2012**, *18*, 999–1002, doi:10.3748/wjg.v18.i9.999.
3. Osman, K.; Kendi, A.T.; Maselli, D. Isolated Angioedema of the Bowel Caused by Aspirin. *Clin J Gastroenterol* **2021**, *14*, 1096–1102, doi:10.1007/s12328-021-01430-6.
4. Turcu, A.F.; White, J.A.; Kulaga, M.E.; Skluth, M.; Gruss, C.B. Calcium Channel Blocker-Associated Small Bowel Angioedema. *J Clin Gastroenterol* **2009**, *43*, 338–341, doi:10.1097/MCG.0b013e31815cf6b9.
5. Alawami, A.Z.; Tannous, Z. Late Onset Hypersensitivity Reaction to Hyaluronic Acid Dermal Fillers Manifesting as Cutaneous and Visceral Angioedema. *J Cosmet Dermatol* **2021**, *20*, 1483–1485, doi:10.1111/jocd.13894.
6. Mousa, O.; Sacco, K.; Wang, M.-H. Losartan-Induced Intestinal Angioedema: 2169. *Official journal of the American College of Gastroenterology | ACG* **2016**, *111*, S1035.

7. Majoni, S.W.; Smith, S.R. Membranous Nephropathy in a Patient with Hereditary Angioedema: A Case Report. *J Med Case Rep* **2008**, *2*, 328, doi:10.1186/1752-1947-2-328.
8. Yang, H.; Wang, W.; Hu, X.; Zhang, X.; Liu, L. Sirolimus-Induced Severe Small Bowel Angioedema: A Case Report. *Medicine (Baltimore)* **2018**, *97*, e12029, doi:10.1097/MD.0000000000012029.
9. Maarek, R.; Sellier, N.; Seror, O.; Sutter, O. Small Bowel Angioedema Due to Intravenous Administration of Gadobenate Dimeglumine. *Diagn Interv Imaging* **2019**, *100*, 459–460, doi:10.1016/j.diii.2019.03.006.
10. Park, S.W.; Bae, I.Y.; Eun, H.W.; Park, H.W.; Choe, J.W. Small-Bowel Angioedema during Screening Computed Tomography Due to Intravenous Contrast Material. *J Comput Assist Tomogr* **2011**, *35*, 549–552, doi:10.1097/RCT.0b013e318224247e.
11. Zvidi, I.; Gal, E.; Rachamimov, R.; Niv, Y. Tacrolimus-Induced Intestinal Angioedema: Diagnosis by Capsule Endoscopy. *Case Rep Gastroenterol* **2007**, *1*, 1–6, doi:10.1159/000104226.
12. A Rare Case of Losartan-Induced Visceral Angioedema - PubMed Available online: https://pubmed.ncbi.nlm.nih.gov/37965705/ (accessed on 24 February 2025).
13. A Rare Cause of Abdominal Pain in Plain Sight - Record Details - Embase Available online: https://www.embase.com/records?subaction=viewrecord&rid=4&page=1&id=L2003268539 (accessed on 24 February 2025).
14. Thalanayar, P.M.; Ghobrial, I.; Lubin, F.; Karnik, R.; Bhasin, R. Drug-Induced Visceral Angioedema. *J Community Hosp Intern Med Perspect* **2014**, *4*, doi:10.3402/jchimp.v4.25260.
15. Ghezzi, C.L.A.; Ghezzi, T.L.; Corleta, O.C. Angiotensin-Converting Enzyme Inhibitors-Induced Angioedema of the Small Bowel Mimicking Postoperative Complication. *Rev Esp Enferm Dig* **2017**, *109*, 481–482, doi:10.17235/reed.2017.4734/2016.
16. Kim, S.H.; Cho, J.Y.; Lim, H.K. CT Findings of Isolated Small Bowel Angioedema Due to Iodinated Radiographic Contrast Medium Reaction. *Abdom Imaging* **1999**, *24*, 117–119, doi:10.1007/s002619900457.
17. Chen, C.-K.; Chang, H.-T.; Chen, C.-W.; Lee, R.-C.; Sheu, M.-H.; Wu, M.-H.; Chou, H.-P.; Shen, Y.-C.; Chiu, N.-C.; Chang, C.-Y. Dynamic Computed Tomography of Angioedema of the Small Bowel Induced by Iodinated Contrast Medium: Prompted by Coughing-Related Motion Artifact. *Clin Imaging* **2012**, *36*, 386–389, doi:10.1016/j.clinimag.2011.10.005.
